# Tensiomyography, functional movement screen and counter movement jump for the assessment of injury risk in sport: a systematic review of original studies of diagnostic tests

**DOI:** 10.3389/fspor.2025.1565900

**Published:** 2025-03-10

**Authors:** Álvaro Velarde-Sotres, Antonio Bores-Cerezal, Josep Alemany-Iturriaga, Julio Calleja-González

**Affiliations:** ^1^ Facultad de Ciencias de la Salud, Universidad Europea del Atlántico, Santander, Spain; ^2^ Departamento de Salud, Universidad Internacional Iberoamericana, Campeche, Mexico; ^3^ Faculdade de Ciências de Saúde, Universidade Internacional do Cuanza Bairro Kaluanda, Cuito, Angola; ^4^ Facultad de Ciencias Sociales y Humanidades, Universidad Europea del Atlántico, Santander, Spain; ^5^ Departamento de Ciencias de Lenguaje, Educación y Comunicaciones, Universidad Internacional Iberoamericana, Arecibo, PR, United States; ^6^ Universidad de La Romana, La Romana, Dominican Republic; ^7^ Department of Physical Education and Sport, Faculty of Education and Sport, University of the Basque Country (UPV/EHU), Vitoria, Spain

**Keywords:** injury prevention, risk factors, functional tests, recovery, assessment

## Abstract

**Background:**

Scientific research should be carried out to prevent sports injuries. For this purpose, new assessment technologies must be used to analyze and identify the risk factors for injury. The main objective of this systematic review was to compile, synthesize and integrate international research published in different scientific databases on Countermovement Jump (CMJ), Functional Movement Screen (FMS) and Tensiomyography (TMG) tests and technologies for the assessment of injury risk in sport. This way, this review determines the current state of the knowledge about this topic and allows a better understanding of the existing problems, making easier the development of future lines of research.

**Methodology:**

A structured search was carried out following the Preferred Reporting Items for Systematic Review and Meta-Analyses (PRISMA) guidelines and the PICOS model until November 30, 2024, in the MEDLINE/PubMed, Web of Science (WOS), ScienceDirect, Cochrane Library, SciELO, EMBASE, SPORTDiscus and Scopus databases. The risk of bias was assessed and the PEDro scale was used to analyze methodological quality.

**Results:**

A total of 510 articles were obtained in the initial search. After inclusion and exclusion criteria, the final sample was 40 articles. These studies maintained a high standard of quality. This revealed the effects of the CMJ, FMS and TMG methods for sports injury assessment, indicating the sample population, sport modality, assessment methods, type of research design, study variables, main findings and intervention effects.

**Conclusions:**

The CMJ vertical jump allows us to evaluate the power capacity of the lower extremities, both unilaterally and bilaterally, detect neuromuscular asymmetries and evaluate fatigue. Likewise, FMS could be used to assess an athlete's basic movement patterns, mobility and postural stability. Finally, TMG is a non-invasive method to assess the contractile properties of superficial muscles, monitor the effects of training, detect muscle asymmetries, symmetries, provide information on muscle tone and evaluate fatigue. Therefore, they should be considered as assessment tests and technologies to individualize training programs and identify injury risk factors.

**Systematic Review Registration:**

https://www.crd.york.ac.uk/PROSPERO/view/CRD42024607563, PROSPERO (CRD42024607563).

## 1 Introduction

Scientific research should be carried out to prevent sports injuries. For this purpose, valid and reliable assessment methods are needed to reduce the number of sports injuries (1–6). As a result of the investigations (3, 5) different methods and technologies have been proposed to assess and identify injury risk factors.

Sports injuries can affect the health and performance of athletes. Therefore, studies and research should be conducted to assess the risk of injury in athletes, thus contributing new knowledge to science (3, 5). In addition, there is a need to preserve the health and well-being of professional players when faced with a high frequency of extremely demanding matches (7). Consequently, strategies must be designed to optimize player availability and minimize factors such as fatigue (7).

These risk factors for sports injuries include characteristics of the athletes, sports and the environment (4). Another factor that has a decisive influence on the probability of suffering an injury as a result of sports practice is the workload (8). Therefore, it is necessary to analyze and study the risk factors that can produce an injury (1–6).

Based on these criteria, there is a need to assess the athlete's risk of injury, taking into account the different intrinsic and extrinsic risk factors that can have a decisive influence on an injury (1, 4). Among the factors to be studied are asymmetries (9), neuromuscular imbalances between limbs (4), muscle stiffness (10, 11), postural control deficits (12, 13) or fatigue (14).

To carry out these analyses, functional tests and muscle assessment methods or technologies are used to detect fatigue, monitor the training load, detect asymmetries or functional imbalances, as possible risk factors for injury (3, 5, 8, 14, 15).

One of the functional tests used to evaluate performance during the vertical jump is the Countermovement Jump (CMJ), as it is a Gold Standard (16). The CMJ is a valid and reliable tool (16) for assessing lower limb power capacity, either unilaterally or bilaterally, as well as detecting asymmetries between limbs.

Similarly, the performance of the CMJ jump on a jumping platform allows the measurement of flight time, contact time, height and power. Starting from this fundamental database, the software designed allows to obtain in real time these parameters linked to the athlete's performance (16). The CMJ can also be used to assess neuromuscular fatigue.

Along with this test, the Functional Movement Screen (FMS) is a valid and reliable tool (12, 13, 17) to assess an individual's fundamental movement patterns. Additionally, this system can be used at the end of the rehabilitation process to help determine if an athlete is ready to return to training. The main purpose of the FMS tool is to identify functional asymmetries and postural or motor control deficits (12, 13).

The FMS is composed of 7 fundamental movement patterns (test), with a numerical value from 0 to 3 according to certain observable markers that require a balance between mobility and stability (12, 13).

Another tool used in the evaluation is Tensiomyography (TMG) is a valid and reliable tool (10, 11, 18, 19) to evaluate the contractile properties of superficial muscles. TMG is a technique to evaluate the mechanical muscle response based on the displacement of the radial muscle belly to a single electrical stimulus (9). As a result of this electrical stimulus, a displacement-time curve is recorded where the following parameters are integrated: maximum radial muscle displacement (Dm), contraction time (Tc), delay time (Td), sustained contraction time (Ts) and relaxation time (Tr) (10, 11, 18, 19).

TMG is a non-invasive tool (11, 18, 19) used to monitor the effects of training during a specific period or throughout the season, to detect bilateral muscle asymmetries, to detect fatigue and to individualize training loads for athletes (11, 18, 19).

Given the existing reality, it is expected to analyse the current technologies, considering the starting existing capacities and the experts in the physical activity and sports, biomechanics and medicine, using the application of the information technology.

Individualized training is key to improving sports performance and preventing injuries. To do so, it is necessary to use new technologies that allow for the assessment of injury risk.

To date, and to the best of our knowledge, there are no previous level studies or evidence 1A demonstrating the use of CMJ, FMS and TMG tool variables for injury assessment.

Therefore, the main aim of this systematic review was to compile, synthesize and integrate international research published in different scientific databases on CMJ, FMS and TMG tests and technologies for the assessment of injury risk in sport. This way, this review determines the current state of the knowledge about this topic and allows a better understanding of the existing problems, making easier the development of future lines of research.

## 2 Methods

### 2.1 Searching strategies

This article is a systematic review focused on the methods of sports injury assessment. This systematic review was carried out following the guidelines of the Preferred Reporting Items for Systematic Review and Meta-Analyses (PRISMA®) (20) guidelines, which helped to improve the integrity. And registered at PROSPERO (ID = CRD42024607563). The methodological issues were solved with the guidance of the Cochrane Handbook for Systematic Reviews of Interventions (21).

The PICOS model was used to determine the inclusion criteria (22): P (Population): “athletes of different sports,” I (Intervention): “injury prevention,” C (Comparators): ““group comparison with multidisciplinary interventions and controls,” O (Outcome): “physical and/or neuromuscular performance measurements, physiological responses, and risk of injury,” and S (study design): “any type of design”.”

A structured search was conducted in MEDLINE/PubMed, Web of Science (WOS), ScienceDirect, Cochrane Library, SciELO, EMBASE, SPORTDiscus and Scopus. The investigation ended on November 30, 2024. Search terms included a mix of medical subject headings (MeSH) and free-text words for key concepts related to assessment methods, high performance athletes and sports injury prevention. Specifically, we used the following search equation: [“injury prevention” (MeSH Terms) OR “injury assessment” (All Fields) OR “sports injuries” (All Fields) OR “injury risk factors” (All Fields)] AND [“Assessment test” (MeSH Terms) OR “TMG” (All Fields)] OR “FMS” (All Fields) OR “CMJ” (All Fields)] AND [“sport” (MeSH Terms) OR “football” (All Fields)] OR “sports” (All Fields) OR “athletes” (All Fields)]. Through this equation, all relevant articles in the field were obtained. The reference sections of all identified articles were also examined by applying the “snowball methods” strategy (23), based on examining the reference sections of the identified articles. All titles and abstracts from the search were cross-referenced to identify duplicates and any potential missing studies (Á.V.-S. and J.C.-G). Titles and abstracts were screened for a subsequent full-text review. The search for published studies was independently performed by two different authors (Á.V.-S. and J.C.-G) and disagreements were resolved through discussions between them.

### 2.2 Inclusion and exclusion criteria

We selected studies providing effectivity results in terms of diagnostic accuracy or diagnostic performance for the different tests used in the assessment of sports injuries were included. The systematic review included original studies of diagnostic tests designs included and systematic reviews, meta-analysis, abstracts of conferences and opinion articles were excluded. In addition, we selected studies that contained a minimum of 10 participants. And for effectiveness studies only those that used at least one technique for the prevention and analysis of sports injuries were considered. The CMJ, the FMS and the TMG were considered as comparison techniques.

For the articles obtained in the search, the following inclusion criteria were applied to final selected studies: (I) studies published in peer-reviewed journals and full text available; (II) the articles examined the effects of sports injury assessment methods; (III) original articles published in peer-reviewed peer-reviewed journals with impact factor; (IV) participants were assessed with the CMJ, FMS or TMG; (V) the study population consisted of athletes; (VI) included the assessment of the risk of injury; (VII) performed on any number or type of athlete regardless of category, experience, competitive level or sex; (VIII) published in English. The following exclusion criteria were applied to the experimental protocols of the investigation: (I) the absence of reliable measurements; (II) studies with less than 10 participants; (III) studies conducted using participants with a previous cardiovascular or musculoskeletal disorder; (IV) studies that will not be performed with athletes; (V) abstracts, non-peer-reviewed papers, and book chapters.

### 2.3 Study selection

Titles and abstracts of publications identified by the search strategy were screened for a subsequent full-text review and were cross-referenced to identify duplicates. All trials assessed for eligibility and classified as relevant were retrieved, and the full text was peer reviewed (Á.V.-S. and J.C.-G). Moreover, the reference section of all relevant articles was also examined using the snowball (23). Based on the information within the full articles, the inclusion and exclusion criteria were used to select the trials eligible for inclusion in this systematic review. Disagreements were resolved through discussions between two authors (Á.V.-S. and J.C.-G).

### 2.4 Data extraction

Once the inclusion/exclusion criteria were applied to each study, the following data were extracted: study source (author/authors and year of publication); population of the sample, indicating the number of participants; sport modality; assessment methods and tests; type of research design; study variables; main findings; characteristics of the intervention; significant differences among the study groups and effects of the intervention.

For each study, we carefully collected information for all eligible publications. Average (±) data and standard deviation (SD) data and size of the sample were extracted from the tables of all the included documents. Subsequently, disagreements were resolved through discussion until a consensus was achieved.

### 2.5 Quality assessment and risk of bias

Methodological quality and risk of bias were assessed by two authors independently (Á.V.-S. and D.M.-J), and disagreements were resolved by third-party evaluation (J.C.-G), in accordance with the Cochrane Collaboration Guidelines (24).

In the Cochrane Risk of Bias tool, the following items were included and divided into different domains: (1) selection bias (items, random sequence generation, allocation and concealment), (2) performance bias (blinding of participants and personnel), (3) detection bias (blinding of outcome assessment), (4) attrition bias (incomplete outcome data), (5) reporting bias (selective reporting), and (6) other bias (other sources of bias).

For each investigation, criteria were shown as “low” if the criteria were fulfilled for a low-risk bias (improbable to severely alter the results) or “high” if the criteria were high risk bias (severely weakening the reliability of the results). If the risk of bias was unknown, it was considered “not clear” (it brings doubts about the results).

The systematic review was based on the established principles by the PRISMA statement (20), a verification list which has as main aim to look for the transparency of the important systematic reviews in the scientific rating of these studies. It has got 27 items and a flow chart with four stages, which includes items considered as essential for the transparent communication of a systematic analysis.

The “Physiotherapy Evidence Database (PEDro)” scale was also used to analyse the methodological quality of all the selected articles. This scale is a tool designed to evaluate the methodological quality of the clinical designs (Table 1) and used in many bibliographic reviews. The aforementioned tool is based on a list developed by Verhagen (25) using the Delphi technique (26).

**Table 1:** “Physiotherapy evidence database (PEDro)” scale to analyse the methodological quality of the studies.

PEDro scale			
1	The criteria of election were specified	Yes	No
2	The subjects were randomly assigned to the groups	Yes	No
3	The assignment was hidden	Yes	No
4	The groups were similar at the beginning in relation to the indicators of prognosis	Yes	No
5	All subjects were blinded	Yes	No
6	All the sports scientists providing therapy were blinded	Yes	No
7	All assessors evaluating at least one of key results were blinded	Yes	No
8	All the measures of at least one of the key results were obtained from more than 85% of the subjects initially assigned to the groups	Yes	No
9	The results of all the subjects receiving treatment or assigned to the control group were given, or when not possible, the data for at least one key result were analysed “in order to treat”	Yes	No
10	The results of statistic comparisons among groups were reported for at least one key result	Yes	No
11	The study provides specific and variability measures for at least one key result	Yes	No

The PEDro scale has got a total of 11 items. Item 1 refers to the external validity of the study, while items 2–9 refer to the internal validity; items 10 and 11 show if the statistic information provided by the authors allows the accurate interpretation of the results. All items in the list are dichotomised as “yes”, “no” or “not reported”. Each “yes” item is given one point, while “no” or “not reported” items do not receive any points at all.

The first item of the PEDro scale was not taken into account in this review, as it was related to the evaluation of the external validity of the studies. Therefore, only items 2–11 were selected for the assessment of the methodological quality. Due to this, the maximum score of an article could not be higher than 10 points, and the minimum, not lower than 0 points.

The evaluation of the heterogeneity was another point to analyse. In this case, we can consider, on the one hand clinical heterogeneity, due to the differences among the types of patients, treatments and endings, and on the other hand, methodological heterogeneity, due to the variability in the designs and bias control.

## 3 Results

### 3.1 Main search

The search on data base reported 510 publications. A digital search was made from sources which generated 65 relevant studies, included in the review. After the detailed review of titles, abstracts and complete articles (60), the publications which fulfilled the criteria of inclusion were a total of 40, in English. A limitation of 15 years of publication was applied. Of the 60 articles included, 5 were excluded due to the fact that they were duplicated. A limitation of 15 years of publication was applied. Of the 60 articles included, 5 were excluded due to the fact that they were duplicated, remaining 55 complete articles for the review. In the last stage of the inclusion of articles, 15 articles were excluded, which were not related to the sports area or which studied different variables.

**Figure 1:**
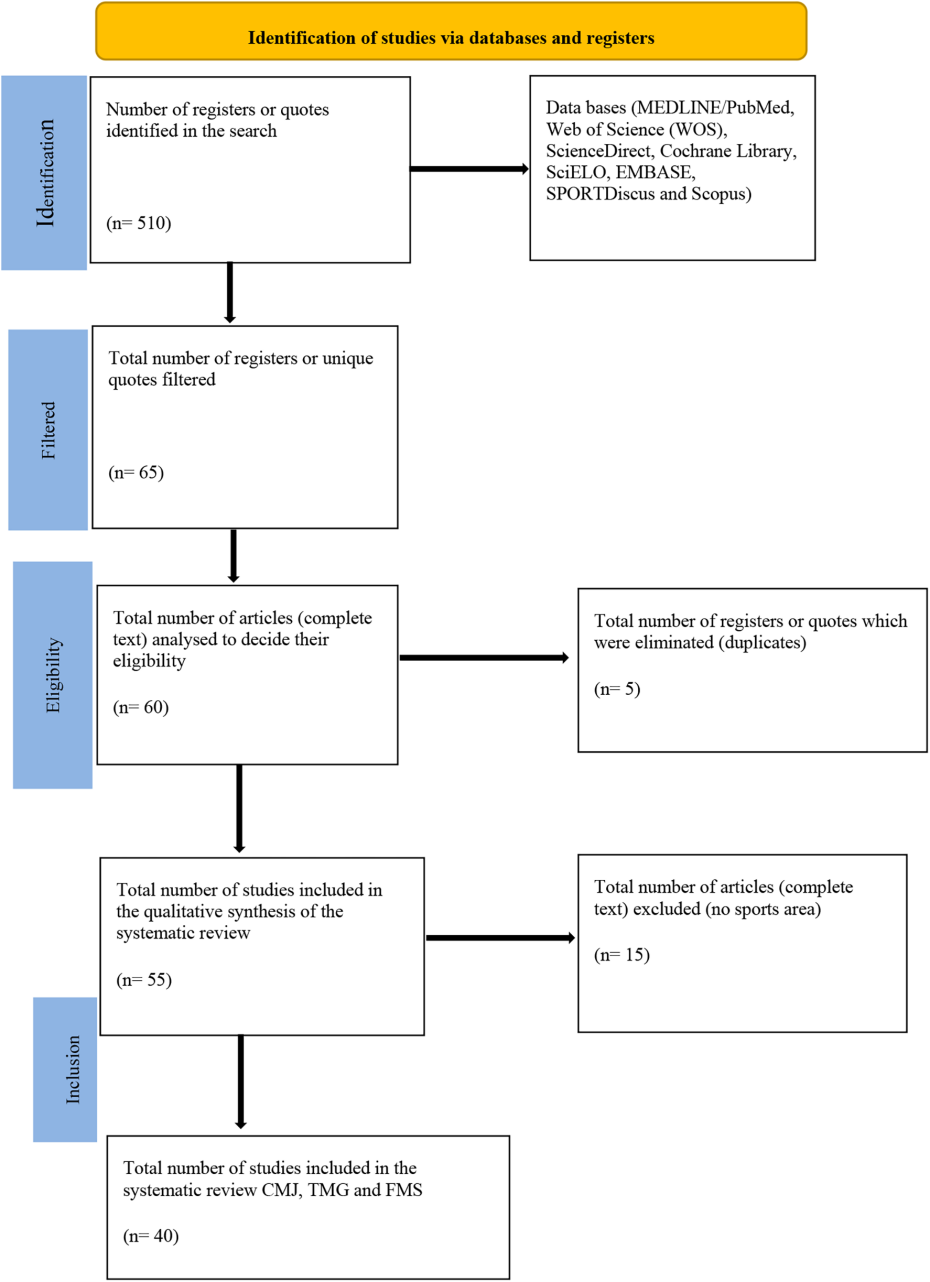
Flow diagram of the study selection.

From the final selection, 40 studies were included. A total of 21 articles were included (27–47) with significative data referring to CMJ, 12 articles (48–59) with significative data referring to the use of FMS, 12 articles (10, 27–30, 46, 60–65) referring to the use of TMG (Figure 1).

### 3.2 Study characteristics

The source of the study (author/authors and year of publication); population of the sample, indicating the number of participants; sport modality; assessment methods and tests; type of research design; study variables; main findings and effects of the intervention are represented on Table 2. 21 articles with significative data referring to CMJ, 12 articles were included with significative data referring to the use of FMS and 12 articles referring to the use of TMG, and Important differences were shown in size, age, gender, design of studies, sport and the evaluation methods used.

**Table 2:** Methodology and results of the interventions.

Study	*N*	Sport	Assessment test	Design	Variables	Main findings	Effects
Li et al. (48)	290	Elite athletes	FMS	Exploratory factor analysis	Internal consistency and factor structure of the 7 tasks	The 7 tasks have got low internal consistency and are not indicative of an only factor. More attention must be paid to the score of each task than to the total score	→
Harshbarge et al. (49)	52	Athletics	FMS	Correlational design	Star Excursion Balance Test, and Balance Error Scoring System	The score for FMS and SEBT asymmetry can show a relation between asymmetry of movement and postural stability. The associations between FMS deep squats and BESS tasks can be related to subjacent neuromuscular control factors.	→
Nicolozakes et al. (50) 2017	38	Football	FMS	Cross-sectional study	Body mass index, body fat percentage	The increase of fat percentage and BMI are related to results in the lower individual FMS.	↑
Duke, et al. (51)	73	Rugby	FMS	Experimental Approach	Injury risk	The quality of movement, analysed by FMS, predicts the risk of injuries by lost time in experienced rugby athletes and it must be considered as an important tool for assessment. Athletes FMS ≤14 have got a significantly higher probability of suffering injuries by lost time in the competitive season.	↑
Yeung et al. (52)	16	Football	FMS	Observational study	Mobility, proprioception, strength	The asymmetry in strength was significant in the prediction of the injury and the FMS score showed a sufficient positive difference.	↑
Chimera et al. (53)	200	Athletics	FMS	Cross-sectional design	Injury History, Sex, and Performance	The injury history and the gender affected the performance of FMS and YBT.	↑
Sannicandro et al. (54)	30	Football	FMS	Correlation study	Asymmetry, Hop Test, Side Hop and Hop Crossover	Better quality movement in FMS, related to a high performance in CMJ and a low percentage of endurance capacity in lower limbs, respectively.	↑
Tous-Fajardo et al. (10)	18	Healthy men	TMG	Observational study	Inter-rater reliability of vastus medialis muscle contractile	The results legitimise the use of TMG for the assessment of the contractual properties of the vastus medialis muscle, particular for Dm and Tc. It is recommended to avoid the quantification Tr and the modifications of IED during many measurements, as it showed an unsatisfactory reliability.	↑
Gil et al. (60)	20	Football	TMG	Correlation study	TMG parameters from rectus and biceps femoris, jumping and sprinting abilities, lateral symmetry	There were no correlations between tensiomyography parameters and power-related motor tasks. In addition, no differences in tensiomyography parameters between dominant and non-dominant legs were found.	↓
Loturco et al. (27)	24	Football	TMG, CMJ	Correlation study	Isokinetic assessments, jump tests, TMG, Asymmetry	Detected asymmetries in the three different methods were not interrelated. Lower-limb asymmetry is not necessarily related to impaired vertical jump performance in soccer players.	↓
García-García et al. (61)	37	Football	TMG	Experimental study	Tc, Dm, Td, knee extensor and flexor muscles	Tc, Td and Dm could be used to individualise the load and intensity of work and to control the effects of the neuromuscular training during the season.	↑
García-García et al. (62)	16	Football	TMG	Observational study	Dm, Tc, Td, Ts, Tr, Vc, muscular asymmetry	It was shown that TMG is a useful way to analyse the neuromuscular characteristics of the players at the beginning of the preseason, and to establish the initial values of the players individually.	↑
Ubago-Guisado et al. (28)	15	Rugby	TMG CMJ	Experimental study	RSA Test, Tc, Dm	The muscular response in the rectus femoris muscle after repetitive sprint actions differs in the different surfaces (sand and grass).	↑
Loturco et al. (29)	41	Athletics	TMG CMJ	A comparative study	Dm, Tc, Td, SJ, reactive strength index	Vertical jump as well as the analysis TMG could be useful to identify and select young athletes.	↑
Rey et al. (63)	31	Football	TMG	Experimental study	Dm, Tc, Td, heart rate and RPE	Significative effects were not found due to the recuperation strategy in the TMG parameters and the perceived muscle pain.	↓
Gonzalo-Skok et al. (31)	30	Basketball	CMJ	A crossover study design	Weight-bearing dorsiflexion test, a modified Star Excursion Balance test	Differences exist between functional movement tests and in jump and/or sprint performance tests between age groups. It could have implications to predict the risk of injury.	↑
Chena et al. (32)	434	Football	CMJ	Correlation study	Body composition, SJ, Abalakov Jump	Besides the biological age and the development of the muscle mass, the position during the game must be taken into account as a relevant variable in the use of the body composition and the performance of the vertical jump as factors of the talent detection.	↑
Menzel et al. (33)	46	Football	CMJ	Correlation study	Lower Limb Asymmetries, isokinetic test	The maximum impulse and strength during CMJ on a strength platform seem to be proper additional variables for the identification of bilateral differences.	↑
Roe et al. (34)	12	Rugby	CMJ	Experimental study	Cycle-ergometer test, performance	The greater weekly changes in CMJ metrics in comparison with CET may indicate differences in the capacities of these tests to measure training-induced lower-body neuromuscular fatigue.	→
Bonato et al. (35)	160	Basketball	CMJ	Cluster randomized controlled trial	Y-Excursion Balance test, lower limb strength, postural control	Including body-weight neuromuscular training in the warm up routines reduced the occurrence of serious injuries in lower limbs in elite female basketball players.	↑
Roche-Seruendo et al. (36)	51	Athletics	CMJ	Experimental study	SJ, Spatiotemporal parameters, muscular performance parameters	The muscular performance parameters do not play a key role in the space-time adaptations experimented by the athletes with higher speed. The authors suggest that the muscular performance parameters would be much more determinant when there is fatigue.	↓
Fort-Vanmeerhaeghe et al. (37)	69	Volleyball and basketball	CMJ	A cross-sectional study	Flight time, jump height, asymmetry	A threshold of 10–15% asymmetry in vertical jump height between limbs can be considered as the physiological norm in basketball and volleyball players.	↑
Fort-Vanmeerhaeghe et al. (38)	29	Basketball	CMJ	A cross-sectional design	Star excursion balance test, sprint test asymmetry	Single leg countermovement vertical jump may be the most useful to predict injury.	↑
Ferioli et al. (39)2018	28	Basketball	CMJ	Experimental study	Fatigue, power	The training period brought a few changes in CMJ, while the capacity to keep DQO repeated efforts was improved. Getting a high session score with loads in the effort training may affect partially and negatively the capacity to produce strength and power.	→
Heishman et al. (40)	10	Basketball	CMJ	Retrospective analysis design	External load, internal stress, fatigue	Omegawave and Catapult technologies provide independent information related to the efficiency and can be useful tools to monitor the performance of the athletes.	↑
Wing et al. (41)	15	Football	CMJ	Experimental study	Strength, power	Superior strength and power qualities have been shown to positively impact successful heading and tackling performance.	↑
Marqués-Jiménez, et al. (42)	10	Football	CMJ	Experimental study	Neuromuscular Fatigue	Internal and external load metrics may allow for predicting the extent of acute fatigue	↑
Fort-Vanmeerhaeghe et al. (43)	81	Young elite team-sports athletes	CMJ	Observational study	Interlimb asymmetries, injury incidence	Athletes with greater interlimb asymmetries, less vertical jump capacity, and lower intermittent aerobic fitness had a greater predisposition to injury	↑
Morgan et al. (55)	45	Students	FMS	Observational Laboratory Study	Interrater Reliability between Raters	The updated FMS has acceptable interrater reliability between minimally, but adequately trained individuals. The updated FMS may be reliably used to assess risk for future injury.	↑
Bernardes Marques et al. (56)	103	Football	FMS	Cross-sectional observational study	Asymmetries	High-performance young soccer players have important functional deficits, especially in tasks involving deep squat and trunk stability, as well as high prevalence of asymmetry between right and left body side.	↑
Dorrel et al. (57)	257	Athletics	FMS	Cross-sectional study	Injury risk	FMS had limited prognostic ability to accurately identify athletes who might be at risk of injury. FMS can be used to assess movement quality.	↓
Fernández-Baeza et al. (64)	27	Football	TMG	A comparative study	Dm, Tc, Td	The variables of TMG (Tc, Dm) inform about the reaction and Tc, which is a key factor in soccer, as well as the muscle tone, to determine if a muscle has a deficit in tone or stiffness.	↑
Oliver Gonzalo-Skok et al. (44)	22	Basketball	CMJ	Experimental study	Power, between-limbs imbalance, bilateral deficit, change of direction.	Training programs substantially improved most of the physical-fitness tests, but only the unilateral reduced between-limbs asymmetry and achieved greater enhancements in actions that mostly required applying force unilaterally.	↑
Ruffieux et al. (45)	33	Volleyball	CMJ	Experimental study	Jump height	For non-professional female volleyball players and a training duration of six weeks, training with a high percentage of CMJ is more effective than one with a high percentage of DJ.	↑
García-García et al. (65)	48	Cycling	TMG	Experimental study	Dm, Tc, Td, Ts	An incremental effort until exhaustion produces peripheral fatigue associated with a decrease in Dm, Tc, Td, Ts, and The Vrd, being more pronounced in biceps femoris than in vastus lateralis and rectus femoris. Coaches can use these changes found in the contractile properties as a reference to detect the muscle fatigue.	↑
Warren et al. (59)	167	Basketball, football, volleyball, cross country, track and field, swimming/diving, soccer, golf, and tennis athletes	FMS	Prospective cohort	Injury, asymmetry	FMS, movement patterns, and asymmetry were poor predictors of noncontact and overuse injury in this cohort of division I athletes.	↓
Smith et al. (59)	19	Healthy men and women	FMS	Observational study	Interrater and intrarater reliability	The results showed that the FMS could be consistently scored by people with varying degrees of experience with the FMS after a 2-hour training session	↑
Huso Paravlic1 et al. (30)	35	Football	TMG, CMJ	Correlation study	Asymmetry	The overall significant, albeit inconsistent, correlations between the diverse performance scores obtained highlight the necessity for a multifaceted and thorough diagnostic strategy in female soccer players.	→
Buoite Stella et al. (46)	23	Football	CMJ, TMG	A cross-sectional observational study	Muscle Asymmetries	Findings suggest an association between lower-limb muscle asymmetries during a dynamic task, such as jumping, and muscle contractile properties evaluated with TMG; moreover, functional asymmetries may be present after ankle injuries.	↑
Delextrat et al. (47)	21	Football	CMJ	Experimental study	Strength-Endurance Training, height	As inadequate eccentric strength and fatigue are both risk factors for hamstring injury, SE training should be considered along with the development of peak eccentric strength, as a component of programs aimed at reducing injury risk in multiple-sprint sports.	↑

↑ positive effect; → no effect; ↓ negative effect; CMJ, counter movement jump; TMG, tensiomyography; FMS, functional movement screen; Dm, maximum radial muscle belly displacement; Tc, contraction time; Td, delay time: Ts, sustain time.

### 3.3 Risk of bias

**Figure 2:**
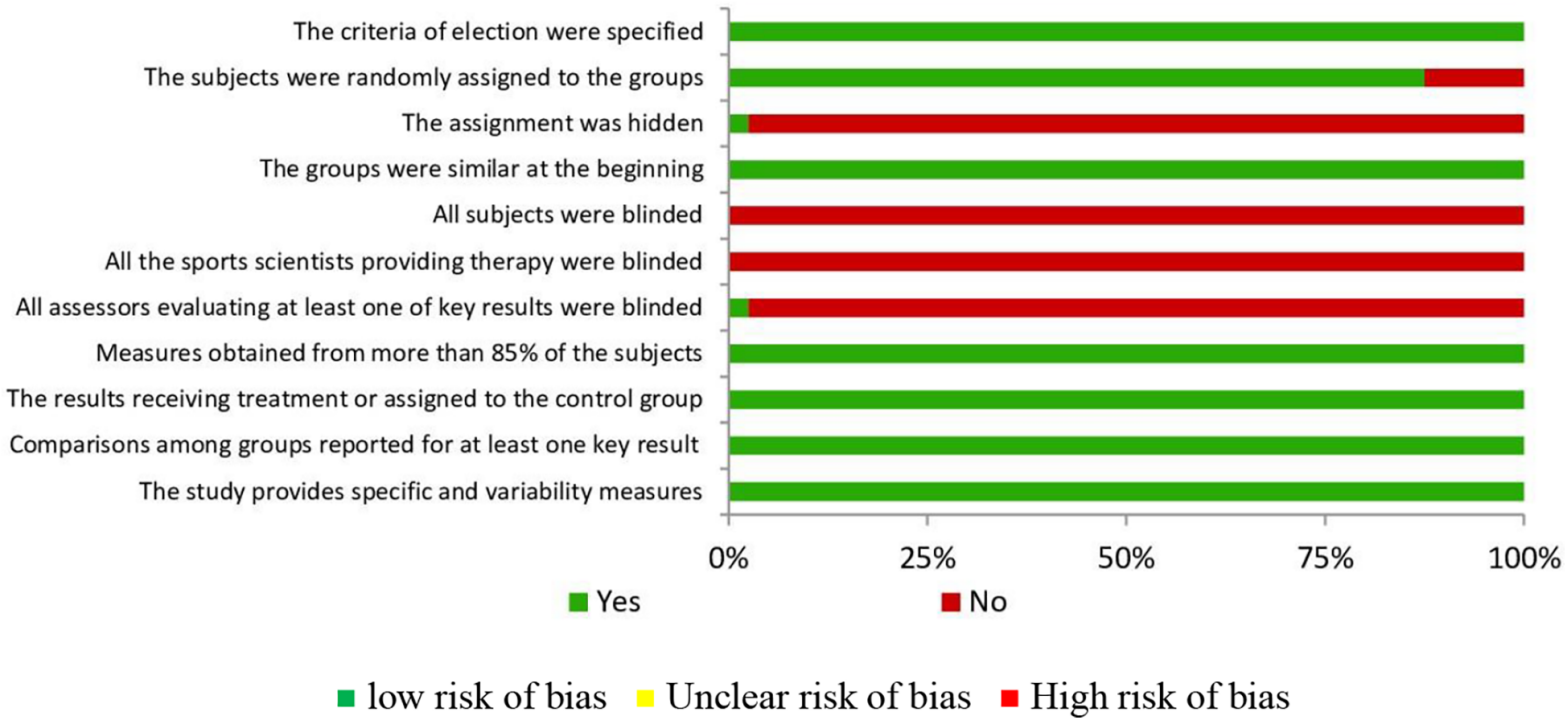
Risk of bias summary: review authors’ judgements about each risk of bias item for each included study.

The methodological quality and the risk of bias were evaluated following the guidelines of the Cochrane Collaboration (24). For each investigation, criteria were shown as “low” if the criteria were fulfilled for a low risk of bias (improbable to severely alter the results) or “high” if the criteria were high risk bias (severely weakening the reliability of the results). If the risk of bias was unknown, it was considered “not clear” (it brings doubts about the results). Every included study was assessed for the risk of bias (24). The full assessments of study quality are shown in Figure 2.

### 3.4 Methodological quality assessment

The methodological quality of the analysed studies varied between 5 and 7 points, with an average of 5.92 points. 33 Articles got 6 points, 5 articles got 5 points and 2 article got 7 points.

Despite the relative heterogeneity of the analysed studies, certain criteria were consistent in all of them. Table 3 shows the criteria which more frequently were obtained belong to item 4, “the groups were similar at the beginning in relation to the most important indicators of prognosis”, to item 8 “the measures of at least one of the key results were obtained from more than 85% of the participants initially assigned to the groups”, followed by item 9 the results of all the subjects receiving treatment or assigned to the control group were given, or when not possible, the data for at least one key result were analysed “in order to treat”, item 10 “The results of statistic comparisons among groups were reported for at least one key result” and 11 “the study provides specific and variability measures for at least one key result”. Finally, just to indicate that none of the studies fulfilled criteria 5 and 6 (subjects and sports scientists were blinded), and only one study fulfilled item number 3 and 7 (the assignment was hidden and all assessors of at least one of the key results were blinded).

**Table 3:** Results according to PEDro scale (*n* = 40).

Clinical trial	1	2	3	4	5	6	7	8	9	10	11	Total
Li et al. (48)	Yes	Yes	No	Yes	No	No	No	Yes	Yes	Yes	Yes	6
Harshbarge et al. (49)	Yes	Yes	No	Yes	No	No	No	Yes	Yes	Yes	Yes	6
Nicolozakes et al. (50)	Yes	Yes	No	Yes	No	No	No	Yes	Yes	Yes	Yes	6
Duke, et al. (51)	Yes	Yes	No	Yes	No	No	No	Yes	Yes	Yes	Yes	6
Yeung et al. (52)	Yes	Yes	No	Yes	No	No	No	Yes	Yes	Yes	Yes	6
Chimera et al. (53)	Yes	Yes	No	Yes	No	No	No	Yes	Yes	Yes	Yes	6
Sannicandro et al. (54)	Yes	Yes	No	Yes	No	No	No	Yes	Yes	Yes	Yes	6
Tous-Fajardo et al. (10)	Yes	Yes	No	Yes	No	No	No	Yes	Yes	Yes	Yes	6
Gil et al. (60)	Yes	Yes	No	Yes	No	No	No	Yes	Yes	Yes	Yes	6
Loturco et al. (27)	Yes	Yes	No	Yes	No	No	No	Yes	Yes	Yes	Yes	6
García-García et al. (61)	Yes	Yes	No	Yes	No	No	No	Yes	Yes	Yes	Yes	6
García-García et al. (62)	Yes	No	No	Yes	No	No	No	Yes	Yes	Yes	Yes	5
Ubago-Guisado et al. (28)	Yes	Yes	No	Yes	No	No	No	Yes	Yes	Yes	Yes	6
Loturco et al. (29)	Yes	No	No	Yes	No	No	No	Yes	Yes	Yes	Yes	5
Rey et al. (63)	Yes	Yes	No	Yes	No	No	No	Yes	Yes	Yes	Yes	6
Gonzalo-Skok et al. (31)	Yes	No	No	Yes	No	No	No	Yes	Yes	Yes	Yes	5
Chena et al. (32)	Yes	No	No	Yes	No	No	No	Yes	Yes	Yes	Yes	5
Menzel et al. (33)	Yes	Yes	No	Yes	No	No	No	Yes	Yes	Yes	Yes	6
Roe et al. (34)	Yes	Yes	No	Yes	No	No	No	Yes	Yes	Yes	Yes	6
Bonato et al. (35)	Yes	Yes	No	Yes	No	No	No	Yes	Yes	Yes	Yes	6
Roche-Seruendo et al. (36)	Yes	Yes	No	Yes	No	No	No	Yes	Yes	Yes	Yes	6
Fort-Vanmeerhaeghe et al. (37)	Yes	Yes	No	Yes	No	No	No	Yes	Yes	Yes	Yes	6
Fort-Vanmeerhaeghe et al. (38)	Yes	Yes	No	Yes	No	No	No	Yes	Yes	Yes	Yes	6
Ferioli et al. (39)	Yes	Yes	No	Yes	No	No	No	Yes	Yes	Yes	Yes	6
Heishman et al. (40)	Yes	Yes	No	Yes	No	No	No	Yes	Yes	Yes	Yes	6
Wing et al. (41)	Yes	Yes	Yes	Yes	No	No	No	Yes	Yes	Yes	Yes	7
Marqués-Jiménez, et al. (42)	Yes	Yes	No	Yes	No	No	No	Yes	Yes	Yes	Yes	6
Fort-Vanmeerhaeghe et al. (43)	Yes	Yes	No	Yes	No	No	No	Yes	Yes	Yes	Yes	6
Morgan et al. (55)	Yes	Yes	No	Yes	No	No	Yes	Yes	Yes	Yes	Yes	7
Bernardes Marques et al. (56)	Yes	Yes	No	Yes	No	No	No	Yes	Yes	Yes	Yes	6
Dorrel et al. (57)	Yes	Yes	No	Yes	No	No	No	Yes	Yes	Yes	Yes	6
Fernández-Baeza et al. (64)	Yes	Yes	No	Yes	No	No	No	Yes	Yes	Yes	Yes	6
Oliver Gonzalo-Skok et al. (44)	Yes	Yes	No	Yes	No	No	No	Yes	Yes	Yes	Yes	6
Ruffieux et al. (45)	Yes	Yes	No	Yes	No	No	No	Yes	Yes	Yes	Yes	6
García-García et al. (65)	Yes	Yes	No	Yes	No	No	No	Yes	Yes	Yes	Yes	6
Warren et al. (59)	Yes	Yes	No	Yes	No	No	No	Yes	Yes	Yes	Yes	6
Smith et al. (59)	Yes	No	No	Yes	No	No	No	Yes	Yes	Yes	Yes	5
Huso Paravlic1 et al. (30)	Yes	Yes	No	Yes	No	No	No	Yes	Yes	Yes	Yes	6
Buoite Stella et al. (46)	Yes	Yes	No	Yes	No	No	No	Yes	Yes	Yes	Yes	6
Delextrat et al. (47)	Yes	Yes	No	Yes	No	No	No	Yes	Yes	Yes	Yes	6

Yes: it presents the studied criterium. No: it does not present the studied criterium.

1. The criteria of election were specified; 2. The subjects were randomly assigned to the groups; 3. The assignment was hidden; 4. The groups were similar at the beginning in relation to the most important indicators of prognosis; 5. All participants were blinded; 6. All the sports scientists providing therapy were blinded; 7. All assessors evaluating at least one of key results were blinded; 8. All the measures of at least one of the key results were obtained from more than 85% of the participants initially assigned to the groups; 9. The results of all the subjects receiving treatment or assigned to the control group were given, or when not possible, the data for at least one key result were analysed “in order to treat”; 10. The results of statistic comparisons among groups were reported for at least one key result; 11. The study provides specific and variability measures for at least one key result.

## 4 Discussion

### 4.1 Summary of main findings

The main aim of this systematic review was to compile, synthesize and integrate international research published in different scientific databases on CMJ, FMS and TMG tests and technologies for the assessment of injury risk in sport. This way, this review determines the current state of the knowledge about this topic and allows a better understanding of the existing problems, making easier the development of future lines of research.

It was decided to carry out a revision of the most relevant bibliography, as well as of the most important published papers, in order to obtain the most outstanding aspects or data to which their authors refer, and this way work in all aspects to be taken into account, during and after the practice of sports, in order to avoid sports injuries.

The indicated measures were verified in terms of efficiency in the different studies analysed in this review. There currently exist many literary proposals which try to gather them in different ways in terms of prevention protocols, studying their effects in a complex way. In spite of this, it was observed that preventive actions are not currently used systematically.

Therefore, the current scientific bibliography describes different methods for the assessment and value of sports injuries, among which CMJ, FMS and TMG stand out.

### 4.2 Counter movement jump (CMJ)

Investigations reveal the validity and reliability of CMJ (16) to assess the power ability of lower extremities either unilaterally or bilaterally. For this reason, the CMJ vertical jump test can be used to monitor athletes' adaptations to training programs through measurements based on flight time, contact time, height and for estimation of lower extremity explosive power (16).

In addition to this, the studies (27, 33, 37, 38, 43, 44) indicate that the CMJ test can be used for the detection of asymmetries. In this regard, impulse and peak power during CMJ on a force platform appear to be additional variables appropriate for the identification of bilateral differences in sports such as basketball, volleyball, or football (33). Therefore, it would be appropriate to calculate the neuromuscular asymmetry of the lower extremities, because a greater neuromuscular asymmetry between legs could lead to a higher incidence of injury. To this end, some studies (37) indicate that a threshold of 10%–15% of vertical jump height asymmetry between limbs can be considered as the physiological norm in players.

As evidenced by (34, 39, 40, 42), CMJ can also be used to assess neuromuscular fatigue. The use of non-invasive strategies to monitor internal stress and external training load can be a valuable tool to identify player fatigue and stress (40). Neuromuscular fatigue can be quantified through CMJ performance, as suggested, its high repeatability and sensitivity proves its usefulness as a fatigue marker (42).

Similarly, the performance of the CMJ jump on a jumping platform allows the measurement of flight time, contact time, height and power. Starting from this fundamental database, the software designed allows to obtain in real time these parameters linked to the athlete's performance (16). Moreover, impulse and maximum strength during CMJ in a strength platform seem to be proper additional variables to identify bilateral differences. Therefore, it is relevant to carry out a vertical jump test in a strength platform to ensure a wide and reliable diagnostic information (33).

In the same way, the studies (41) support the idea that strength and power training is important for performance. It has also been indicated that CMJ training is more effective than drop jump training in improving jump height in female volleyball players (45). In addition, it has been shown that the inclusion of neuromuscular body mass training in warm-up routines can reduce the incidence of serious lower extremity injuries (35).

With respect to the risk of injury, some studies (43) indicate that athletes with greater asymmetries, lower vertical jump capacity and lower intermittent aerobic fitness have a greater predisposition to injury. Therefore, it is recommended to monitor CMJ and asymmetries given their sensitivity to detect significant differences between injured and healthy young athletes (43).

Therefore, the quantification of neuromuscular deficits through the CMJ is essential to identify individuals who may be at risk of injury (38). In addition, inadequate eccentric strength and fatigue are risk factors for injury (47). Therefore, eccentric strength development should be considered as a component of programs aimed at reducing the risk of injury (47).

### 4.3 Functional movement screen

Results show the use of the FMS to evaluate the quality of fundamental movement patterns, identify an individual's limitations or asymmetries as a potential risk factor for injury. This way, the studies (12, 13) show that 7 exercises (deep squat, hurdle step, lineal lunges, shoulder mobility, active straight leg raise, flexion in trunk stability and rotational stability) with a score of 0, 1, 2 and 3, allows evaluation of an athlete's basic movement patterns, mobility and stability.

In addition, the FMS could be used for asymmetry detection in athletes (58). Regarding asymmetry, some studies (52) have shown a significant difference between injured and non-injured professional football players, indicating that asymmetry could be used as a possible identifier of injury risk and has been found to be negatively associated with lower extremity injuries. This is also evidenced by relating the FMS to other assessment tests, such as the Star Excursion Balance Test (SEBT), and Balance Error Scoring System (BESS) scores (49). In this regard, it has been suggested (49) that associations between the FMS asymmetry score and the SEBT composite score may indicate a relationship between movement asymmetry and postural stability.

Also, the internal consistency and factorial structure of the 7 tasks of the functional movement test in elite athletes have been studied (48). In this regard, the results (55) of an updated version of the FMS indicate that it has acceptable inter-rater reliability among individuals with minimal but adequate training. The updated FMS can be used reliably to assess the risk of future injury (55). In addition, results have shown (59) that the FMS could be consistently scored by individuals with varying degrees of experience with the FMS after a 2-h training session. In a controlled laboratory study (66) with the FMS, intra-rater reliability was found to be strong and appears to be strengthened when individuals have experience using the FMS in addition to clinical experience.

In the same way, the different scores of FMS can be narrowly related to the athlete's height, weight, BMI and body fat percentage (50). As some studies show (50), the increase of body fat percentage and BMI is related to results in lower individual FMS, which prove potentially poor movement patterns in bigger athletes. Furthermore, other variables such as injury history or gender may influence performance on FMS tests (53). In this regard, Lower global FMS scores have been reported in athletes with a history of injury or surgery (53).

Finally, related to the results, many investigations (51, 55–57, 67) show that FMS ≤14 athletes have got a significantly higher probability of suffering injuries. In this regard, it is shown that participating subjects with scores ≤14 have a significantly higher probability of injury compared to those with higher scores.

Therefore, FMS could be used to assess the movement quality of athletes or active adults (66), with the aim of improving the movement pattern, which could reduce a risk factor for future injuries (51, 57, 58), so it should be considered as an assessment tool.

### 4.4 Tensiomyography

Studies show TMG as a valid and reliable (10, 11, 18, 19, 29) assessment tool to evaluate the contractile properties of superficial muscles. TMG is a technique to evaluate the mechanical muscle response based on the displacement of the radial muscle belly to a single electrical stimulus (10). As a result of this electrical stimulus, a displacement-time curve is recorded where the following parameters are integrated: maximum radial muscle displacement (Dm), contraction time (Tc), delay time (Td), sustained contraction time (Ts) and relaxation time (Tr) (10, 11, 18, 19).

In addition, TMG is shown to be a non-invasive method (11, 18, 19), which can be used to monitor the effects of training during a specific period or throughout the season, to detect muscle asymmetries in soccer players, basketball players and athletes (27, 30, 44, 46), lateral symmetry between dominant and non-dominant legs (60), provide information on muscle tone (64) and to detect fatigue (61).

Despite the reliability shown by this method, studies indicate that it is necessary to thoroughly follow a previously fixed protocol (10, 29, 46, 62–64) for each individual evaluation. In this sense, the recording of the radial displacement will be performed on the muscle belly after an external electrical stimulus (29). For this, the point of placement of the sensor must be taken into account, the placement of two adhesive electrodes, the duration of the electrical stimulus must be standardized at 1 millisecond of duration, of increasing intensity according to the protocol used, with varying intensity (50, 75 and 100 mAp) (68) and the recovery (periods of 10 s) between each electrical stimulus must be established (29, 64, 68).

Finally, it is important to highlight the results of the assessment of the variables TC and Dm in the biceps femoris and in the rectus femoris. As the studies (10, 64) conclude that the use of TMG for the evaluation of the contractile properties of the muscle, particularly for Dm and Tc, they can be an indicative to individualise the load and intensity of work. Therefore, TMG data (29, 61, 62, 64) can be used to individualize training programs, the intensity, to monitor the effects of neuromuscular training throughout the season and adjust the training load.

### 4.5 Strengths, limitations, and future lines of research

Our scientific research on a systematic review of original studies of diagnostic tests evidences the importance of conducting an assessment to identify the different risk factors for injury and to individualize training programs.

The sport modality, the sample, the terminology, the way to classify sports injuries and the technologies used, as it usually occurs in all the studies of similar characteristics, can be considered as limitations, as the election of some implies the rejection of others which could provide other type of data of wide interest.

The review could be biased when the bibliographic research was only carried out in classified magazines, having been rejected some published interventions which could have fulfilled the rest of the fixed requisites to be included.

It should be pointed out that the conclusions provided by our review have been carried out according to the articles found by our search strategy and selected under our eligibility criteria; therefore, there always exists the probability that there are studies which because of classification problems or search limits have not been included in this systematic review.

Future research should investigate the effectiveness of tests and assessment technologies for use in the injury rehabilitation process. Future studies should also investigate the effectiveness of a wider range of assessment technologies and test, which allow for the identification and detection of injury risk, as well as fatigue monitoring. Further studies are needed to evaluate the effectiveness of assessment technologies to individualize recovery.

## 5 Practical applications

We must note the importance of the assessment through valid and reliable technologies to identify the different injury risk factors. The CMJ can be used to assess lower limb power capacity unilaterally or bilaterally, to monitor athletes' adaptations to training programs through measurements based on flight time, contact time and height. In addition, it can be used to detect asymmetries, indicating that a threshold of 10%–15% of vertical jump height asymmetry between limbs can be considered the physiological norm in players, and to assess neuromuscular fatigue. FMS can be used to assess the quality of fundamental movement patterns and identify an individual's limitations as a potential risk factor for injury, through 7 exercises with a score of 0, 1, 2 and 3. Thus, the assessment of the different results which use FMS, shows that the participants with scores ≤14 have a significantly higher probability of injury compared to those with higher scores. TMG can be used for muscle assessment, particularly using Tc and Dm variables, with a variable intensity protocol (50, 75 and 100 mAp), and periods of 10 s between consecutive measurements, allowing individualization of training programs. Furthermore, inadequate strength and fatigue are risk factors for injury, so eccentric strength development should be considered as a component of programs designed to reduce injury risk. Therefore, quantification of neuromuscular deficits is essential to identify individuals who may be at risk for injury.

## 6 Conclusions

The results of this systematic review of the different studies presents the evidence of the technologies CMJ, FMS, and TMG, for the assessment of sports injuries. The CMJ vertical jump allows us to evaluate the power capacity of the lower extremities, both unilaterally and bilaterally, detect neuromuscular asymmetries and evaluate fatigue. Likewise, FMS could be used to assess an athlete's basic movement patterns, mobility and postural stability. Finally, TMG is a non-invasive method to assess the contractile properties of superficial muscles, monitor the effects of training, detect muscle asymmetries, symmetries, provide information on muscle tone and evaluate fatigue. Therefore, they should be considered as assessment tests and technologies to individualize training programs and identify injury risk factors.

## Data Availability

The original contributions presented in the study are included in the article/supplementary material, further inquiries can be directed to the corresponding author/s.
